# Automated Reconstruction of Three-Dimensional Fish Motion, Forces, and Torques

**DOI:** 10.1371/journal.pone.0146682

**Published:** 2016-01-11

**Authors:** Cees J. Voesenek, Remco P. M. Pieters, Johan L. van Leeuwen

**Affiliations:** Experimental Zoology, Department of Animal Sciences, Wageningen University, Wageningen, Netherlands; University at Buffalo, SUNY, UNITED STATES

## Abstract

Fish can move freely through the water column and make complex three-dimensional motions to explore their environment, escape or feed. Nevertheless, the majority of swimming studies is currently limited to two-dimensional analyses. Accurate experimental quantification of changes in body shape, position and orientation (swimming kinematics) in three dimensions is therefore essential to advance biomechanical research of fish swimming. Here, we present a validated method that automatically tracks a swimming fish in three dimensions from multi-camera high-speed video. We use an optimisation procedure to fit a parameterised, morphology-based fish model to each set of video images. This results in a time sequence of position, orientation and body curvature. We post-process this data to derive additional kinematic parameters (e.g. velocities, accelerations) and propose an inverse-dynamics method to compute the resultant forces and torques during swimming. The presented method for quantifying 3D fish motion paves the way for future analyses of swimming biomechanics.

## Introduction

Quantification of swimming kinematics is essential to perform biomechanical analyses of fish locomotion. Kinematic parameters, such as tail-beat frequency or amplitude [1, 2], are frequently used to express changes in swimming behaviour. More detailed descriptions of the fish motion are required to perform mechanism-oriented studies into propulsion and manoeuvring, using for example computational fluid dynamics techniques [3, 4]. Many types of fish motion are essentially three-dimensional, as fish are free to move through the water column to explore their environment, escape predators, or hunt for prey. Unless relatively rare cases of single-plane motion are considered, it is necessary to quantify swimming kinematics in three dimensions.

Experimental data on swimming motion are commonly obtained in the form of high-speed videographs [5–7], from which the motion and/or kinematic variables are extracted. Historically, this has often been done by manual digitisation [6, 8–10]. This process is tedious, time consuming and may introduce a user-dependent bias, e.g. the results may differ consistently between individual digitisers. An automated approach is therefore preferred, allowing higher data throughput and consistency compared to manual digitisation.

Automated fish tracking methods have been proposed in 2D [11, 12], but the assumption of two-dimensional motion restricts application to a narrow range of swimming behaviour. A method was developed [13] that allows tracking of multiple fish in three dimensions. However, it does not support rolling motion, and describes the fish centreline with relatively low accuracy using a quadratic polynomial in lateral direction, and a quartic polynomial in longitudinal direction. To our knowledge, an automated method to reconstruct arbitrary 3D motion (translation, pitch, roll and yaw) and body curvature with sufficiently high accuracy in all variables to analyse dynamics (i.e. forces and torques) is still lacking.

Resultant forces and torques can be reconstructed from kinematic data, under specific assumptions. This approach, commonly known as inverse dynamics, has been used often for terrestrial and aerial locomotion [14, 15], but it has rarely been applied to analyse fish swimming. Resultant forces and torques were computed for swimming zebrafish larvae in 2D [16]. Other studies [17, 18] compute swimming forces and bending moments along the fish body, assuming (simplified) fluid-dynamic models. This requires assumptions to be made on the fluid motion, that may not hold for low and intermediate Reynolds number flow regimes, or complex manoeuvres with strong vortex interactions [19]. We calculate the resultant 3D forces and torques on the body directly from the motion of the fish, only assuming a mass distribution based on its shape. To our knowledge, three-dimensional tracking has never been used to calculate resultant forces and torques to study the dynamics of fish swimming and manoeuvring.

In this article, we describe a method that allows accurate tracking of the fish’s body position, orientation and deformation in 3D space, and reconstruction of resultant forces and torques from high-speed video sequences from two or more arbitrary viewpoints. We use an optimisation procedure to minimise differences between a parameterised model fish and fish silhouettes from segmented high-speed video frames. The method is validated using synthetically generated data, and demonstrated on three-camera synchronised high-speed video of a three day old zebrafish larva.

## Materials and Methods

**Fig 1:**
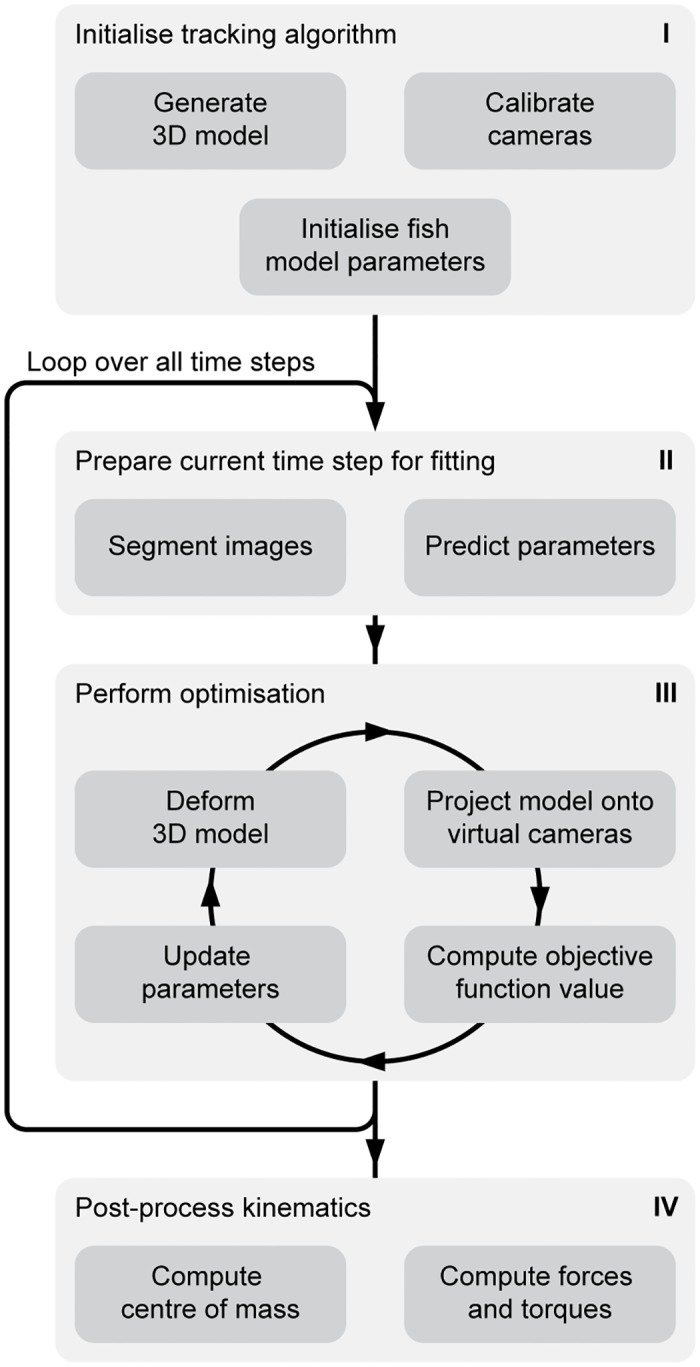
Schematic overview of the tracking method. We provide initial information by creating a 3D model of the fish, calibrating the cameras and initialising the fish position by clicking the snout and tail position in the images from every camera (I). For every time step, we segment the fish in the images from all cameras and predict the fish position, orientation and body curvature based on previous frames (II). The predicted parameters are then used to initialise an optimisation algorithm. This algorithm finds the set of parameters (body curvature, position and orientation) that minimises the difference with the high-speed video frames (III). Once the optimisation has been performed for all frames, we compute the centre of mass and, by inverse dynamics, the resultant forces and torques over time (IV).

Our tracking method, outlined in Fig 1 and implemented in MATLAB (R2013a; version 8.1, The Mathworks, Natick, Massachusetts, USA), is based on the creation of an *in silico* representation of the videography experiment: we create a virtual, parameterised fish and project it onto virtual cameras. We find the fish’s position, orientation and deformation by minimising the difference between the virtual and the actual images using an optimisation algorithm. We post-process these kinematics to yield resultant forces and torques on the body, and other quantities of interest. For a full mathematical treatment of the methods, we refer to S1 Appendix.

### Creating the parameterised fish model

To represent the fish *in silico*, we create a three-dimensional virtual representation of the fish. To this end, we construct a 3D model of the outer surface of the fish. The motion and deformation of this model is parameterised based on the typical motion of body and caudal fin swimmers.

We assume that: (1) The fish bends its body in lateral direction only [13, 16, 20], resulting in a single deformation plane. (2) Transverse sections remain perpendicular to the deformed centre line; only pure bending is applied to the fish model. This allows us to describe the body deformation by a single parameter: the curvature along the centre line [16]. (3) There is no passive or active deformation of the medial fin fold relative to the body. Because the fin fold has a small mass compared to the trunk of the fish, its deformation will only be a minor contribution to the resultant forces and torques from inverse dynamics. (4) The motion of the pectoral fins is ignored in the present version of the 3D fish tracker. For zebrafish larvae, the pectoral fins have been suggested to play a minor role in propulsion during slow swimming [7], and often remain adducted during fast swimming [21]. (5) The 3D position and orientation (roll, pitch, yaw) of the head are completely unconstrained.

**Fig 2:**
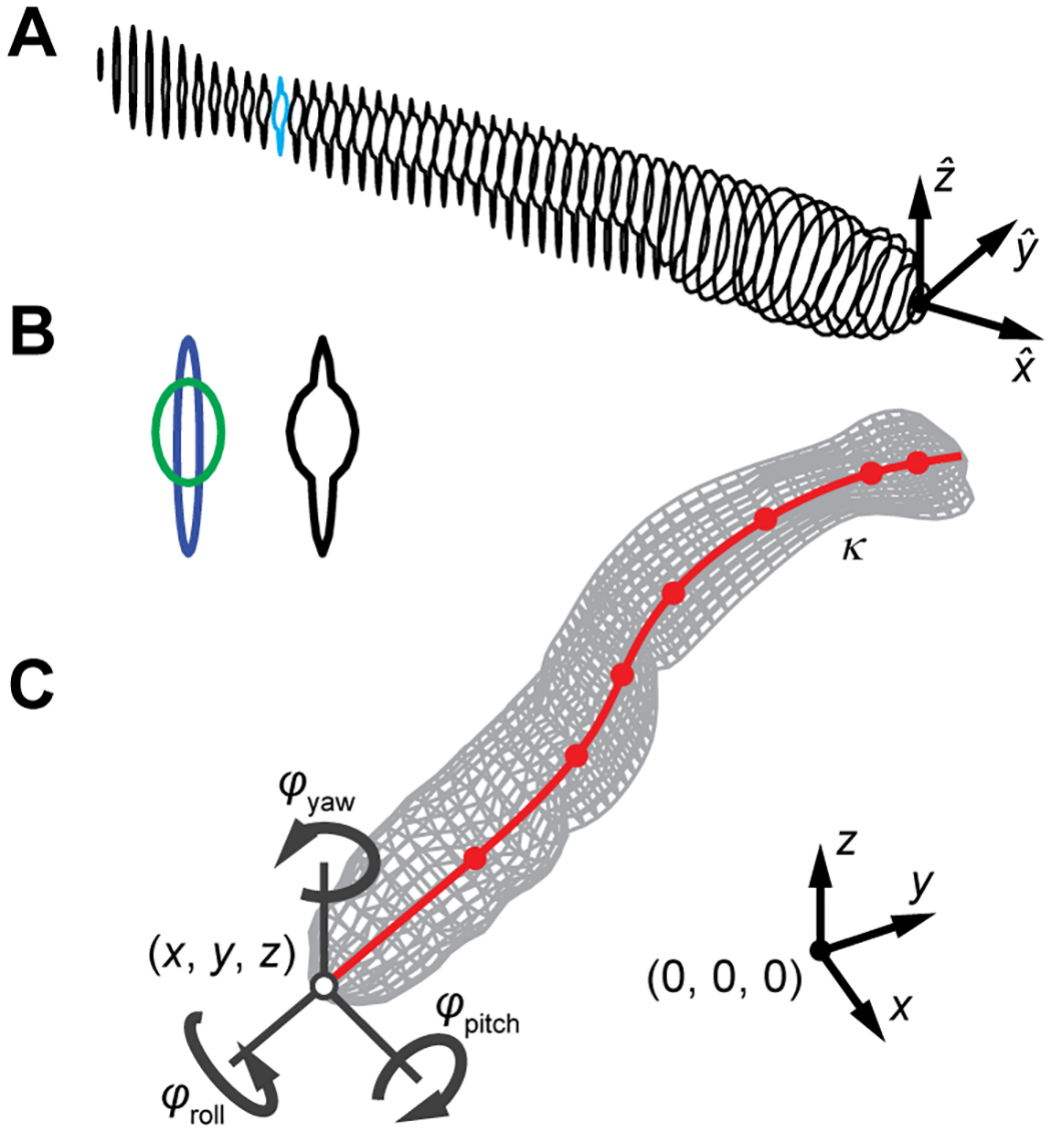
Construction of the *in silico* zebrafish. (A) A series of cross-sectional shapes is combined into a three-dimensional body model. The cross-section indicated in blue is shown in more detail in (B). (B) Generation of cross-section in the tail region: two “components”, body (green) and fin (blue), with different cross-sectional shapes are merged into a single cross-section (black). (C) Parameterisation (position, head orientation and body curvature) of the three-dimensional body model. The position of the tip of the snout is described by the coordinates *x*, *y*, *z*, and head orientation by the three Tait-Bryan angles *φ*_roll_, *φ*_pitch_, *φ*_yaw_. Body deformation is parameterised by prescribing a curvature *κ* along the centreline (red) at a number of control points, indicated by the dots; the body surface is deformed along with the centreline, under the assumption that the transverse sections stay plane, and perpendicular to the centreline.

We describe the three-dimensional surface of the fish by a longitudinal series of transverse sections [16] (see Fig 2A). This approach allows large flexibility in the body shapes to be described, and makes it relatively easy to create new three-dimensional surface models. Furthermore, deforming the body model under the assumption of pure bending becomes a simple matter of rotating and translating cross-sections.

For our case of the larval zebrafish, we distinguish three “components”: trunk, eyes and medial fin fold. The cross-sectional shape of these components is described separately by respectively cubic splines, superellipses (generalised ellipses with an exponent ≠ 2) and ordinary ellipses. To determine the parameters of each cross-sectional shape at different points along the body, we photograph zebrafish larvae laterally and dorsally using a digital camera (DP50, Olympus, Japan) mounted on a microscope (Stemi SV11, Zeiss, Germany), and then digitise these photos using a custom MATLAB R2013a program.

At each point along the fish, the three components are merged into a single cross-section by finding their outermost contour (see Fig 2B). All merged cross-sectional shapes are generated with the same number of circumferential points, allowing us to connect them into quadrilateral faces and thus create a 3D surface model. Because we assume that cross-sections remain plane and perpendicular to the fish’s centreline, we can easily deform the model based solely on the centreline position.

The head motion is described by its three-dimensional position and orientation (roll, pitch, yaw). This also prescribes the orientation of the plane in which the fish bends. The body deformation is described by the local body curvature along the centre line in this deformation plane. We reconstruct curvature rather than position or local angle to allow applications of the method to use higher order spatial derivatives. We prescribe the curvature to be zero in the stiff head region of the fish, where we expect no deformation (the anterior 10% of a larval zebrafish). We define a relatively small number of control points (7 for the model of the zebrafish larva) where curvature is prescribed, which we interpolate to all nodes of the high-resolution body model using a cubic spline. This reduces computational load during tracking, while enabling us to capture arbitrary curvature distributions along the fish.

We calculate the deformed surface of the fish from a set of parameters (i.e. head position and orientation, and body curvature) and the 3D model (see Fig 2C). First, we calculate the deformed shape of the body based in a coordinate system attached to the head, with the *x*-coordinate in caudo-rostral direction. The interpolating spline fit describes the curvature along the body as a third-order piecewise polynomial, which we integrate analytically to obtain the local angle of the centreline. The shape of the centreline is constructed by rotating each subsequent segment by this local angle. Each cross-section is then translated with the centreline, and rotated by the local angle to create the deformed surface model.

This model is subsequently rotated in 3D space by multiplication with a rotation matrix, created using the roll, pitch and yaw angles. Finally, it is translated to the specified snout position. The result is a three-dimensional surface representing the fish shape for the prescribed set of parameters.

### Pre-processing video frames

The main input for the tracking algorithm is a set of high-speed video images of a swimming fish from two or more cameras. In order to track the fish, these images must be segmented into fish silhouette pixels and background pixels. The result of this procedure is a binary image, with background pixels set to 0 and fish pixels set to 1.

The specific implementation of the segmentation procedure is strongly dependent on the video setup. We shortly outline the procedure for our example case of larval zebrafish, which was implemented in MATLAB R2013a. First, we correct the video sequence for intensity fluctuations originating from the incandescent light sources. We obtain a correction factor for these fluctuations by calculating the average background intensity, and normalising it by its maximum value. Because the zebrafish larvae have translucent fins with low greyscale contrast to the background, we cannot use intensity thresholding. Instead, we calculate the magnitude of the spatial gradient, threshold this at a specified value and fill all holes; this results in a white silhouette of the fish on a black background.

### Fitting the body model

The goal of the tracking algorithm is to find the set of fish model parameters that corresponds best to the set of video frames. To assess how well a set of parameters matches a set of video frames, we compare a simulated image of the fish to the segmented video frames. This comparison can be expressed in a single scalar, that is low for a good fit, and high for a bad fit.

To find the set of fish parameters that minimises this number at every time step, we use an optimisation algorithm. However, we cannot directly apply the optimisation to the goodness-of-fit term, because the problem of reconstructing a three-dimensional shape from a small number of viewpoints is ill-posed. A unique solution may not exist and the optimal solution may vary strongly with small changes in the projections. We therefore introduce a regularising term that ensures smoothness of the body curvature, and thus reduces the risk of local minima with unrealistic body curvature. The regularised objective function *f*_tot_ is therefore given by

$$f_{\text{tot}}\left(\Omega \right)=f_{\text{GoF}}\left(\Omega \right)+f_{\text{reg}}\left(\Omega \right),$$ (1)

where **Ω** is the set of fish parameters, *f*_GoF_ is the term expressing the goodness of fit and *f*_reg_ is the regularising term. The solution that minimises this function is selected to represent the state of the fish in the current time step.

The goodness-of-fit term is computed by determining the overlap between projections of the virtual fish and the segmented high-speed video images in the current time step. We generate these projections with virtual cameras at calibrated positions and orientations of the experimental cameras (using a bundle adjustment approach for the zebrafish experiment). The projections of the three-dimensional body model onto the virtual image planes are overlayed onto the segmented video images. We count all non-zero pixels in this combined image, and subtract the number of overlapping non-zero pixels. The total number of pixels that differ between all virtual-actual image pairs is our goodness-of-fit term.

The regularising term is defined to become larger if the body curvature is less “smooth”; this suppresses sets of parameters with unrealistically steep curvature gradients. We compute this term by integrating the squared curvature gradient along the fish, weighted with a function that is high near the head and small near the tail:

$$f_{\text{reg}}=\int_{0}^{1}w\left(s\right){\left(\frac{\text{d}\kappa (s)}{\text{d}s}\right)}^{2}\;\text{d}s,$$ (2)

where *s* is a normalised parameter along the fish midline, *w*(*s*) is the weighting function of the form *c*_0_ *e*^−*c*_1_*s*^ with constants *c*_0_ and *c*_1_, and *κ*(*s*) is the local curvature. Due to the weighting function, the regularisation penalises curvature gradients near the head more strongly than near the tail, corresponding to the expected deformation of the fish. The final objective function is computed by summing the goodness-of-fit term and the regularising term.

The objective function Eq (1) is minimised using the Nelder-Mead (or downhill simplex) algorithm [22, 23], which marches an *N*-dimensional simplex with *N* + 1 vertices through optimisation space to find an optimal set of parameters. We initialise the algorithm by extrapolating from the solution in previous time steps, or from a manual indication of the snout and tail tip in the first time step. A rough optimisation is performed from this initial solution, after which we restart the optimisation with ten-fold reduced tolerances. If the objective function differs more than a threshold value from the value in the previous frame, additional optimisations are performed from a randomised initial condition near the previous solution, until the difference is sufficiently small. Applying this procedure to all time steps, we get a description of the state of the fish in terms of the model parameters for the complete video sequence.

### Post-processing and inverse dynamics

The result from the fish tracker is a time series of head positions, head orientations and body curvatures, sampled at every frame in the high-speed video. These data can be post-processed to compute derived quantities of interest (velocities, accelerations, forces, and torques) to answer biological questions on the mechanics of swimming.

Computing these derived quantities involves the computation of (second) derivatives, requiring smoothing of the solution to ensure accurate results. We apply a penalised least squares approach [24] to the raw model parameters—no further smoothing is applied to the derived quantities. A fourth order derivative regularisation term is used, ensuring that all second time derivatives are smooth [25]. The smoothing parameter is chosen by visual evaluation of the resulting derived quantities, such that unrealistic high-frequency components disappear and relevant low-frequency information is retained. The derivatives are computed of the smoothed data using second order finite differences.

The resultant force on the fish body can be computed from the centre of mass (CoM) acceleration and total fish mass. Computation of both quantities requires knowledge of the mass distribution. By assuming a constant density (of water) of the fish volume, the surface description can be used as a mass distribution. We triangulate the optimised surface, and compute the CoM position and fish mass with a method for general polyhedra [26]. We calculate the acceleration of the CoM by double differentiation of its 3D position, yielding the resultant force vector by multiplication with the fish mass according to Newton’s second law. This approach reconstructs the resultant of all external fluid forces acting on the body. Note that the added mass of the surrounding water contributes to these fluid forces, and does not have to be implemented explicitly.

Calculating the resultant torque on a rigid body can be done in a similar manner to the force reconstruction: it is equal to the moment of inertia multiplied by the angular acceleration. However, for the case of a swimming fish this is not applicable, since a significant portion of its mass is moving over distances of the same order of magnitude as its body size, causing the moment of inertia to vary significantly [16]. We have developed an alternative approach, where we calculate the total angular momentum of the body by an extension of a previously developed method for calculation of moments of inertia [26]; see the S1 Appendix for the mathematical background. Taking the time derivative of the total angular momentum L yields the resultant torque **τ** of a three-dimensional, arbitrarily shaped, deforming body:

$$\tau =\rho \;\frac{\text{d}}{\text{d}t}\left\{\underset{V}{\;\int \int \int \;}r^{*}\left(x\right)\times v^{*}\left(x\right)\;\text{d}V\right\},$$ (3)

where *ρ* is the fish’s mass density, r* is the distance vector from the CoM and v* is the velocity vector relative to the CoM.

To simplify interpretation of the calculated forces and torques, we express them in a local coordinate system (*x*_fish_, *y*_fish_, *z*_fish_), aligned with the deformation plane of the fish and moving with its CoM. In the deformation plane, *x*_fish_ is aligned with the resultant body angle [16], which is defined as a local moment of inertia-weighted average angle. Torque vectors in this coordinate system can be interpreted as being respectively “roll”-, “pitch”-, “yaw”-torques. To provide a more intuitive definition of the “forward” force, we define it in the direction of the CoM velocity vector, in addition to the fish coordinate system.

### Larval zebrafish kinematics setup

As an example, we created high-speed videography of a three days post fertilisation zebrafish larva (*Danio rerio* Hamilton 1822). We used a three-camera setup, with one vertical camera (pco.dimax HS4, PCO, Kelheim, Germany; 2000 × 2000 pixels, 75 μs exposure, 500 pixels along the 3.5 mm fish), and a left- and right-facing camera (respectively FASTCAM APX RS and FASTCAM SA5, Photron, Tokyo, Japan; 1024 × 1024 pixels, 33 μs exposure, 250 pixels along the 3.5 mm fish) at 30° to horizontal. A macro lens at f/2.8 (105 mm AF Micro-NIKKOR f/2.8D, Nikon, Tokyo, Japan) with 27.5 mm extension tubes was used on all three cameras. The cameras were synchronised with a digital delay pulse generator (9618+, Quantum Composers, Bozeman, Montana, USA), running at 2000 pulses (frames) per second. Approximately 50 larvae were placed in a regular-hexagonal tube (16 mm sides, 80 mm long), positioned such that the optical axes of all cameras were perpendicular to the air-glass-water interface. Parallel light, created by shining a fibre optic cold light source (KL 150 B, Schott AG, Mainz, Germany) through a 250 mm lens (52 mm 250D close-up lens, Canon, Tokyo, Japan), was aligned with the camera axis. This allows for the creation of high contrast shadow images with large depth-of-field (approximately 8 mm for 15 × 15 mm field of view) at shutter speeds in the order of 50 μs. These experiments were approved by the animal ethics committee of Wageningen University.

### Test cases

#### Motion verification

**Fig 3:**
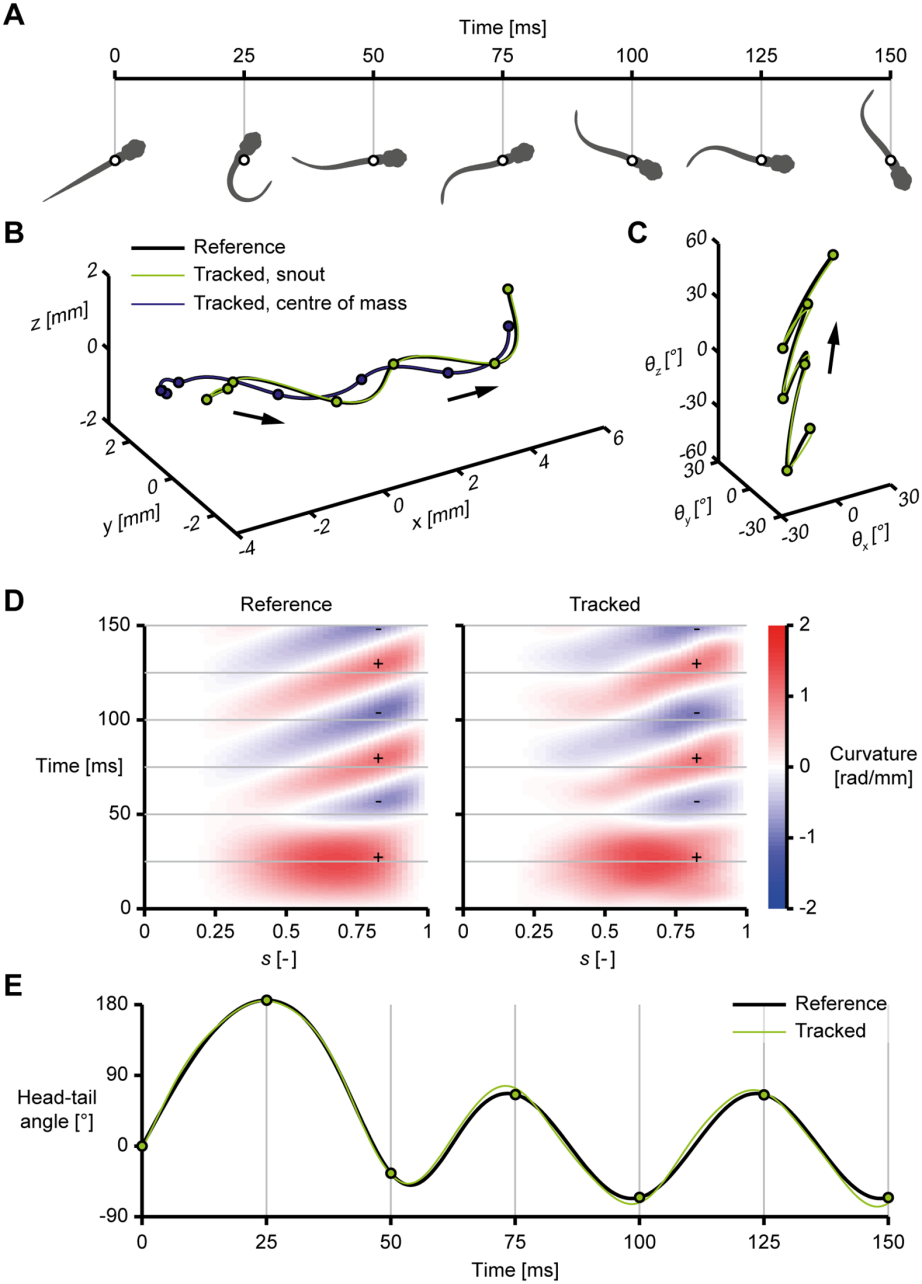
Results of tracking a simulated swimming fish. (A) Projections in the world *x*, *y*-plane of the analytically prescribed simulated swimming motion. (B) Path of the snout and the centre of mass, reference (thick, black) and tracked (thin, respectively green and purple), the arrow indicates the direction in time. The dots correspond to the fish states depicted in (A). (C) The rotation angle vector over time, reference (thick, black) and tracked (thin, green), the arrow indicates the direction in time. (D) Body curvature (colours) as a function of normalised position along the body *s* (horizontal axis) and time (vertical axis) for the reference (left) and tracked result (right), *s* = 0 corresponds to the head, *s* = 1 corresponds to the tail. The grey lines correspond to the fish states depicted in (A). (E) Head-tail angle for the reference (thick, black) and tracked (thin, green) solution. This is effectively the difference in angle between the first and last point on the body, and is the net result of the curvature in every location along the body. The grey lines correspond to the fish states depicted in (A).

In order to assess the motion tracking accuracy of the method, we generated a sequence of images simulating a swimming fish with an exactly known motion. We prescribed the snout motion, orientation and the body curvature analytically, such that it has comparable properties (i.e. spatiotemporal resolution, angle amplitudes, velocities, curvature amplitudes) to actual zebrafish swimming kinematics. A C-start-like motion is prescribed first, which then smoothly blends into a “continuous” swimming mode with a travelling body wave along the body (see Fig 3A).

We projected the prescribed shape of the body model onto three simulated cameras, oriented similarly to the set we used for the larval zebrafish. We simulated a frame rate of 2000 frames per second, and set all virtual cameras to have a field of view of 15 × 15 mm at 1024 × 1024 pixels, resulting in approximately 340 pixels along the simulated fish (*ℓ* = 5 mm). The projections were then Gaussian blurred (*σ* = 1 pixel) and given a 45% decreased contrast. We generated normally distributed (*σ* = 5% of maximum intensity) additive noise at a 10× lower resolution than the images (102 × 102 pixels), oversampled this to full resolution and Gaussian blurred it (*σ* = 5 pixels). This noise image was added to the generated images to simulate dirt and other disturbances in the background of the image. We created synthetic images at two other resolutions: 512 × 512, and 2048 × 2048 (respectively 170 and 680 pixels along the fish) with the same kinematics and camera settings as used for the motion verification.

#### Inverse dynamics verification

In order to verify the validity of the approach for computing forces and moments from a triangulated surface, we simulated a cylinder to which we applied prescribed forces and torques. We generated a cylinder with a diameter of 1.25 mm and a length of 5 mm and applied simple prescribed forces and torques. We integrated the forces over time using trapezoidal integration (MATLAB’s trapz), we integrated the torques using the midpoint rule [27, 28]. The resulting translation and rotation were applied to the three-dimensional, triangulated cylinder model and fed directly into the inverse dynamics module of the post-processor.

## Results

### Motion verification

**Fig 4:**
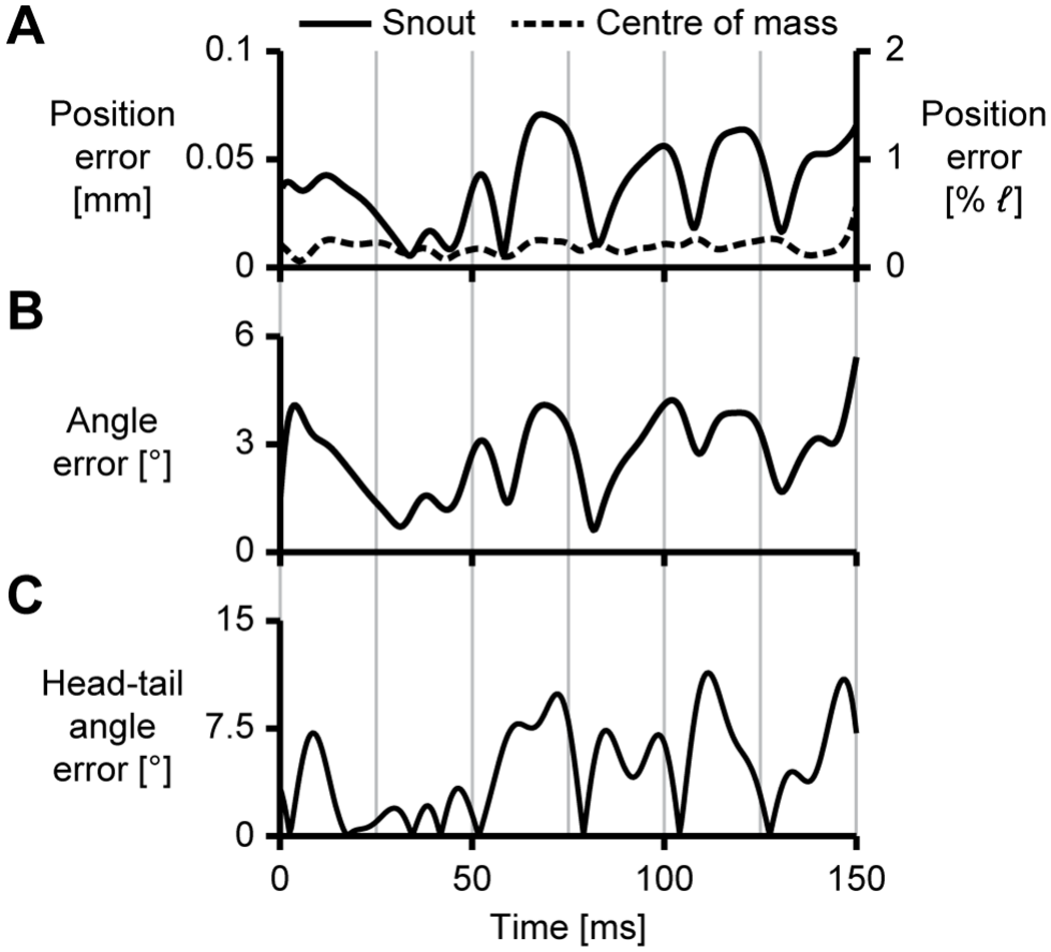
Errors in tracking of a simulated swimming fish, corresponding to the results shown in Fig 3. The grey lines correspond to the time points indicated in Fig 3A. (A) Magnitude of the error in snout (solid line) and centre of mass (dashed line) position, expressed by the Euclidean distance between the reference and tracked points. (B) Magnitude of the angle error vector of the head, expressed by the distance between the tips of the reference and the tracked rotation axis-angle representation. (C) Error in head–tail angle.

We tracked the synthetically generated images (see section Test cases) to assess how accurately the motion is reproduced by the tracker (see Figs 3 and 4). The snout position was tracked with an error smaller than 2% body length over the entire motion (see Figs 3B and 4A). The centre of mass position is reconstructed more accurately, with an error smaller than 0.5% over the image sequence. The snout rotation angle was reproduced with a magnitude of the error vector less than 6° (see Figs 3C and 4B). The curvature is shown in Fig 3D: the largest deviation of the tracked curvature from the reference values occurs near the head. We assess the accuracy of the body curvature reconstruction by computing the integrated value over the entire body—effectively the difference in angle between the first point on the head and the last point on the tail. This head–tail angle is reconstructed with an error smaller than 11°, approximately 6% of its maximum value (see Figs 3E and 4C).

### Inverse dynamics verification

**Fig 5:**
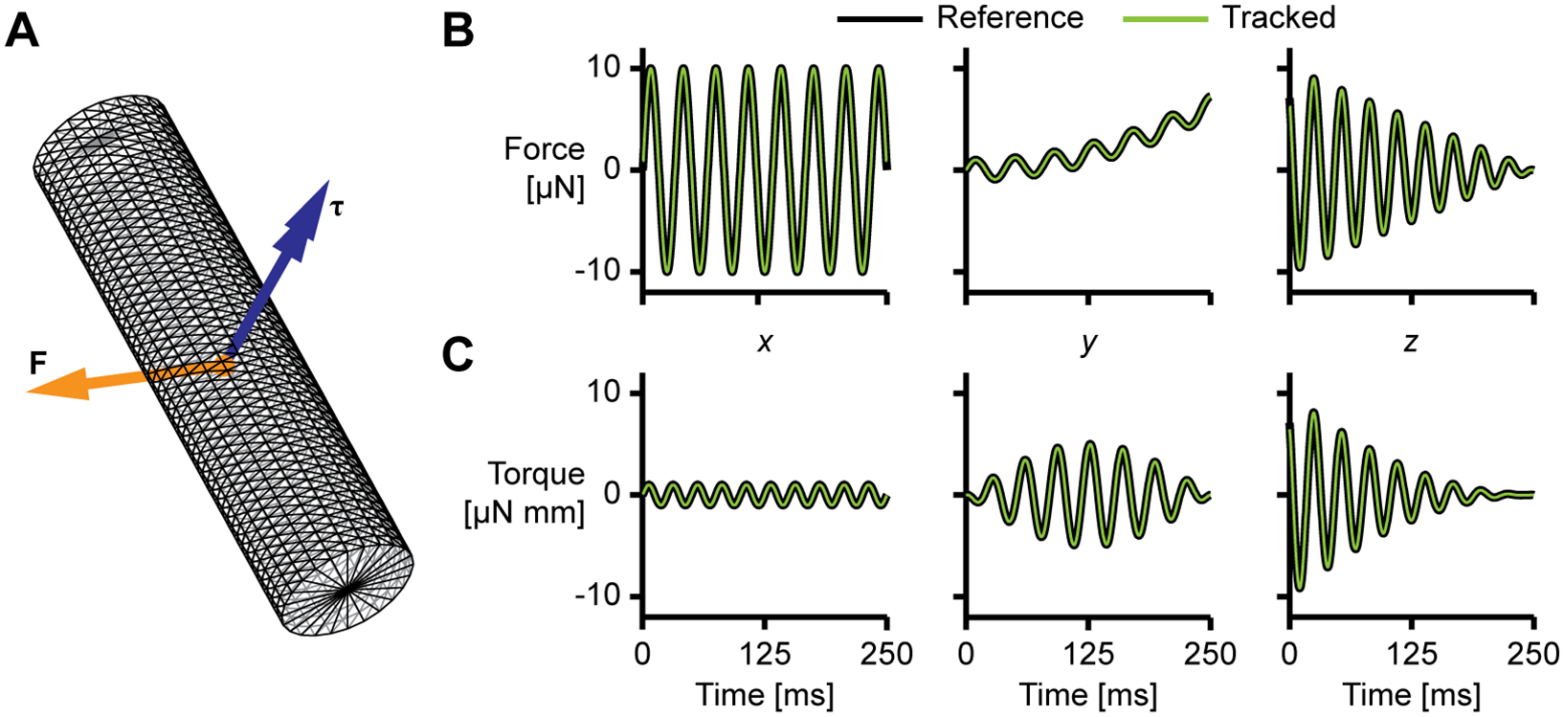
Results of the inverse dynamics algorithm on a simulated rigid body. (A) Triangulated cylinder surface with prescribed time-dependent force and torque. (B) Applied force: reference (thick, black) and computed (thin, green), from left to right *x*-, *y*- and *z*-components. (C) Applied torque: reference (thick, black) and computed (thin, green), from left to right *x*-, *y*- and *z*-components.)

The results in Fig 5B and 5C verify that our inverse dynamics method functions correctly in principle—all components of the reconstructed forces and torques are practically identical to the prescribed reference values.

**Fig 6:**
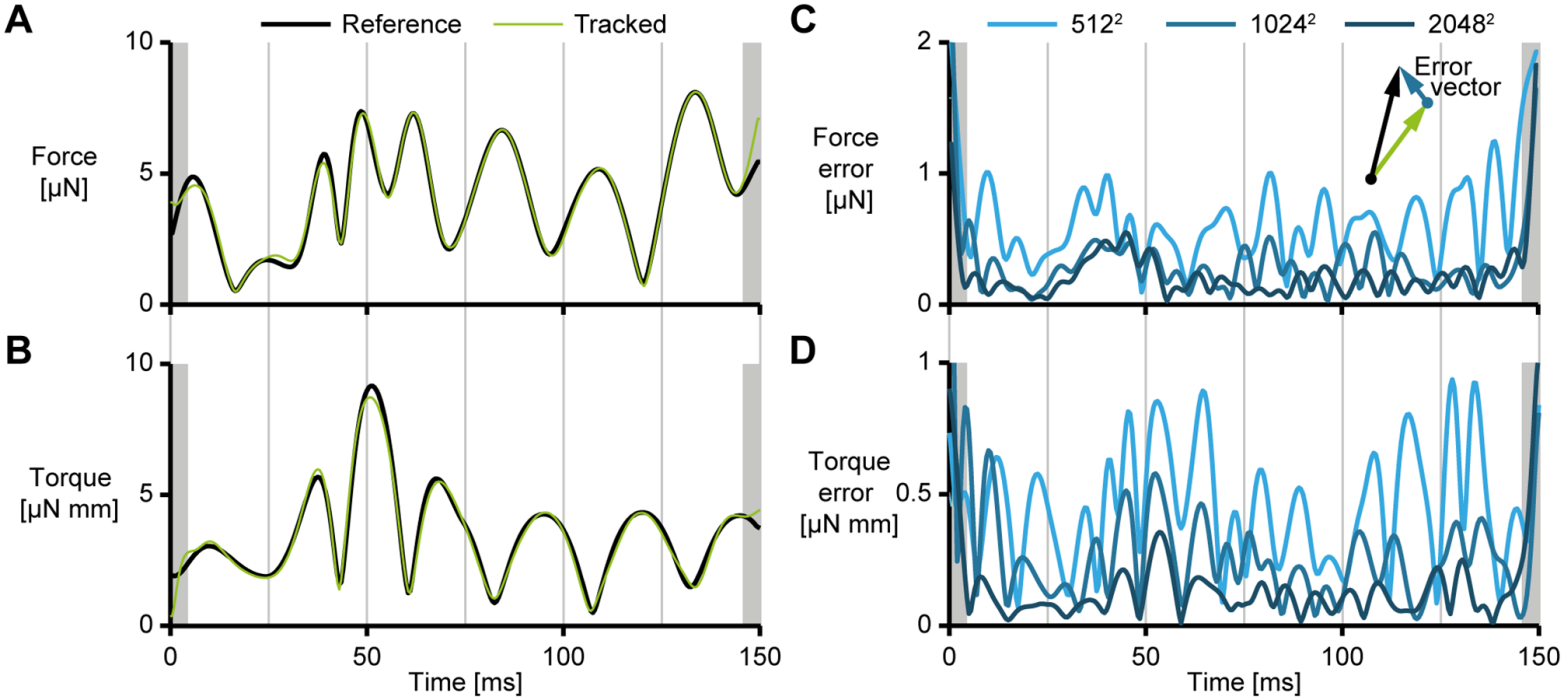
Inverse dynamics results of the simulated fish from Fig 3. The reference forces and torques were computed by applying our inverse dynamics method to the prescribed triangulated body shape. The grey lines correspond to the time points indicated in Fig 3A. (A) Resultant force on the fish, reference (thick, black) and tracked (thin, green) at a resolution of 1024 × 1024 pixels. (B) Resultant torque on the fish, reference (thick, black) and tracked (thin, green) at a resolution of 1024 × 1024 pixels. (C) Magnitude of the force error vector ||**F**_ref_ − **F**_calc_|| and (D) the torque error vector for three resolutions of the generated images: 512 × 512 (light blue), 1024 × 1024 (medium blue) and 2048 × 2048 (dark blue) pixels, respectively approximately 170, 340 and 680 pixels along the fish. The grey bands indicate the first and last 5 frames that have reduced accuracy due to edge effects and may be cut off.

A final step in the verification is the reproducibility of forces and torques from kinematics sequences tracked from video. We fed the prescribed three-dimensional body shape directly into the inverse dynamics module to compute reference forces and torques. We also tracked the generated images and computed the forces and torques from these results (see Fig 6). Having verified the inverse dynamics method with the moving cylinder, we consider the first as our reference and compare it to the latter.

In general, there is strong agreement between the reference and tracked solutions, with a maximum error of approximately 0.5 μN in force and 0.5 μNmm in torque for the solution with a resolution of 1024 × 1024. The dependency on resolution is as expected: higher resolutions will give more accurate results since the body mass distribution can be reconstructed with higher fidelity. The largest deviations occur in the first and last frames, due to edge effects in the smoothing and numerical differentation. Fish swimming data will generally consist of two types of sequences: starts and “continuous” swimming. Edge effects for starts can be eliminated by assuming zero time derivative for all kinematic parameters in the first frame, and cutting off the last few frames. Edge effects for “continuous” swimming can be eliminated by cutting off a few frames from the start and end of the movie sequence during post-processing. Hence, for fish swimming in general, edge effects should not present a major problem for solution accuracy.

### Analysis of three-dimensional fast start of a larval zebrafish

**Fig 7:**
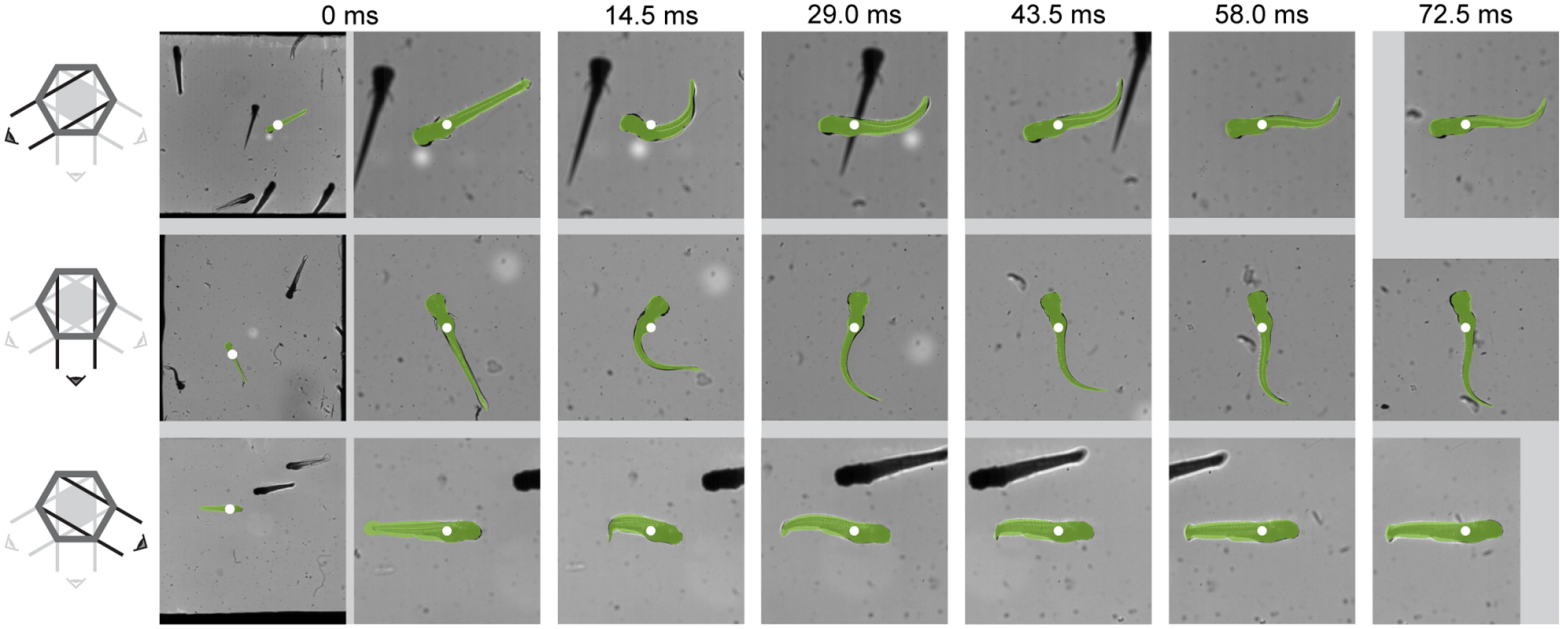
Overlap between the tracked fish and the video images of a three days post fertilisation zebrafish larva. The tracked fish (green) is overlayed over high-speed shadow images for a fast-start of a three days post fertilisation zebrafish larva, with its centre of mass indicated by white dots. Each row shows data from a different camera, from top to bottom oriented at: 30° to horizontal from the left, vertical, and 30° to horizontal from the right, as illustrated on the left hand side of each row. The first frame (0 ms) is shown in full, and zoomed in to the fish to illustrate the field of view size; the rest of the frames are only shown zoomed in.

Fig 7 shows an example tracked result of a fast-start of a zebrafish larva three days post fertilisation (see also S1 Video). The difference between the fish model and the high-speed images is small in all three views, also in the presence of optical occlusions in the form of other fish (around 29 ms). Deviations occur mostly near the head, where the body curvature is underestimated.

**Fig 8:**
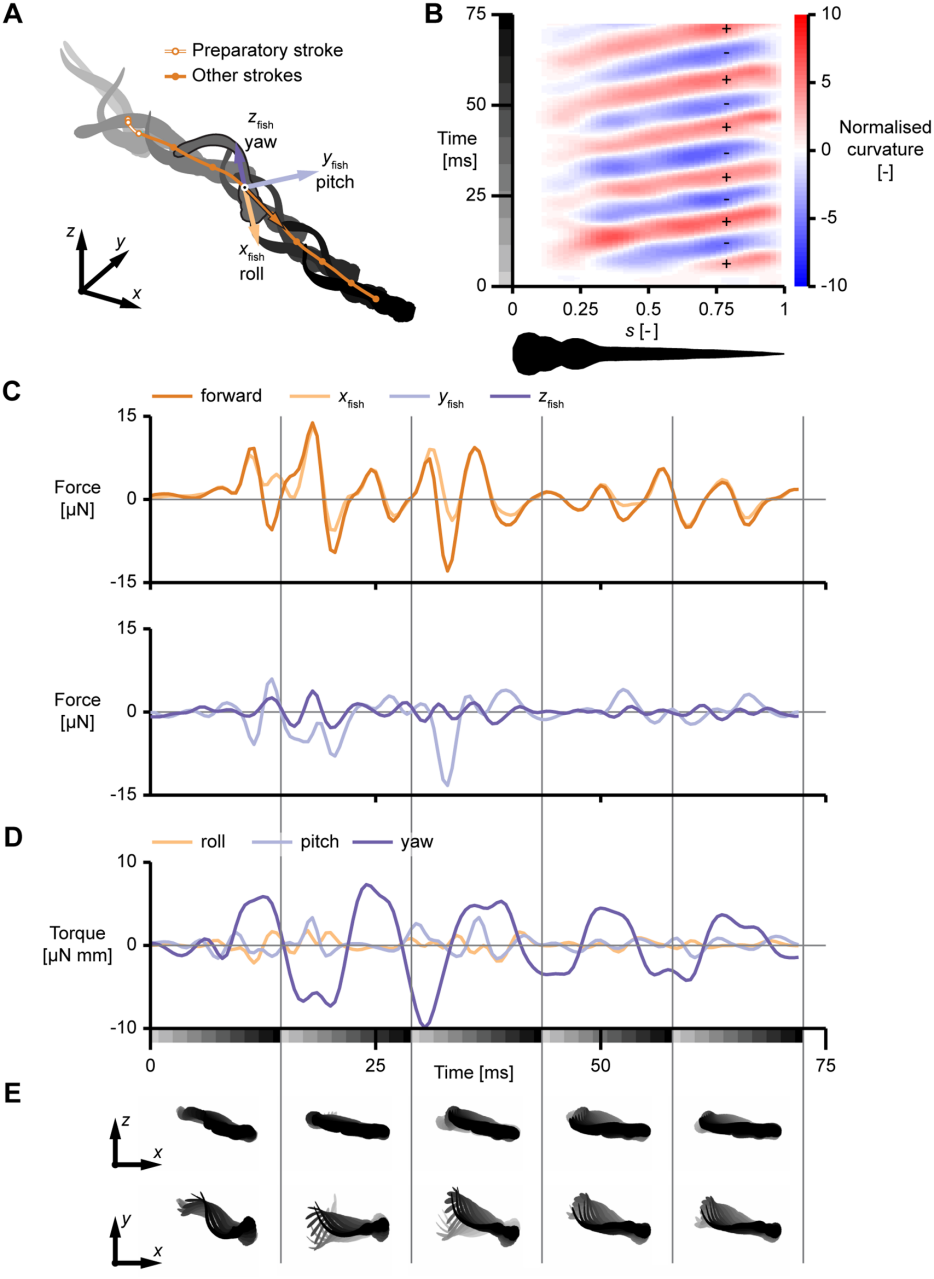
Post-processing result for a fast-start of a three days post fertilisation zebrafish larva. (A) Tracked fish model, time is shown by fill colour (from light grey to black), the path of the centre of mass is indicated by the orange line, with circles corresponding to each of the depicted fish shapes. The preparatory stroke is indicated by a narrow white line. The axes (*x*, *y*, *z*) define the world coordinate system, the axes (*x*_fish_, *y*_fish_, *z*_fish_) define an instantaneous local coordinate system for the highlighted fish shape, defined in the deformation plane of the fish aligned with an inertia-weighted average of the local deformation angle. (B) Local body curvature (colours) along the fish length (horizontal) and time (vertical). The grays on the time axes correspond to the fish shapes in (A). (C) Resultant force on the body in “forward”, *x*_fish_-, *y*_fish_- and *z*_fish_-direction, in respectively dark orange, light orange, light purple and dark purple. The “forward”, *x*_fish_- and *y*_fish_, *z*_fish_-components are shown separately for reasons of clarity. (D) Resultant torque on the body in *x*_fish_-, *y*_fish_- and *z*_fish_-direction, in respectively light orange, light purple and dark purple. The grayscale boxes on the time axes correspond to the fish shapes in each section in (E). (E) Time trace of the body shape in the world *x*, *y*- and *x*, *z*-plane (from light grey to black) for each of the 5 time-slices in (D) and (E), every second frame is shown.

The post-processed result for the same movie is shown in Fig 8. During the preparatory stroke, the CoM moves very little, after which it increases its speed and moves along a waving path (Fig 8A). The motion is not confined to a single plane: the fish moves downward by approximately 1.4 mm over a total distance of 8.3 mm. The curvature (Fig 8B) shows clear waves along the body after the start, moving from approximately 0.25 ℓ to close to the tail. The curvature of the highly flexible tail is mainly caused by a strong interaction with the surrounding fluid, resulting in a complex pattern.

The resultant three-dimensional forces and torques (Fig 8C and 8D) start at low amplitudes until the tail starts moving at high speed at the end of the preparatory phase. The forces in sideward and upward direction oscillate around zero, resulting in a path that is approximately straight. In forward direction, there is a mainly positive force during the start, indicating that the fish is accelerating. After the start, the average force per cycle is around zero, indicating an approximately constant cycle-averaged speed. The pattern of produced force looks non-periodic, similar to earlier results for zebrafish larvae [16]. Slight differences from periodicity at the level of position will result in large differences in the force time series.

The torque about the upward axis (“yaw”) is largest over most of the motion compared to the “roll”-torque and “pitch”-torque, because most of the fish’s mass is rotating in its deformation plane. For the examined tail beats, the yaw torque shows a double-peaked pattern, similar to earlier observations for zebrafish larvae [16]. Though smaller than the yaw torque, the roll and pitch torques are considerable, mainly causing pitch (up to approximately 15°) and an oscillating roll angle (up to approximately 30° during the start).

This example demonstrates that the tracker performs well on high-speed video images of swimming fish. Furthermore, it illustrates that swimming behaviour is essentially three-dimensional, and needs to be analysed as such.

## Discussion and perspectives

Testing of the method using synthetically generated data shows that our tracking method reconstructs position, orientation, curvature, and forces and torques accurately. The example data with a three day old zebrafish demonstrate that it is applicable to real-world high-speed videography.

The accuracy of the extraction of the fish silhouette from the high-speed images strongly determines the fidelity of the result. The quality of segmentation is influenced by image contrast and spatial resolution. Higher image resolution will in general lead to a higher fidelity of reconstruction, both in motion and forces and torques. Higher contrast between fish and background will lead to a sharper silhouette, resulting in smaller tracking errors.

An important source of error is the regularising term that we use to suppress non-physical solutions. It cannot distinguish between spurious and physical curvature gradients, and will suppress any strong gradient, regardless of its source. Most importantly, this leads to an underestimation of curvature in the region near the stiff head section. However, reducing the penalty below a certain threshold may lead to unrealistically steep changes in curvature in the same region. These errors have a limited effect on the reconstructed mass distribution, since the local error can be compensated by changing body curvature elsewhere. Therefore, resultant forces and torques will still be accurate.

For some cases, our assumptions on the fish deformation may not hold; the fish body may twist, the medial fin fold may deform in a more complex way [29], and subtle out of plane movements may be used to generate the fluid dynamic torques that roll and pitch the animal. Furthermore, the assumption that transverse cross-sections remain perpendicular to the centreline may be invalid for fish with a low aspect ratio body shape, unlike the slender zebrafish larvae. These effects may influence hydrodynamic force generation, but will have a minor influence on the accuracy of the mass distribution of the fish. Since we use the mass distribution directly to calculate forces and torques, they are sufficiently accurate under the present assumptions.

The assumed morphology may not match perfectly with the measured fish and will therefore result in errors. We generate body models separately from the experiments, as opposed to previous approaches [11, 13], which generated a body model from the images themselves. We chose our morphology-based approach to maximise use of *a priori* information on the 3D shape of the fish, which cannot be perfectly reconstructed from only three camera views. To prevent tracking errors, care should be taken that the chosen body model corresponds well to the target fish by e.g. generating separate shape models for each individual fish. In our case, we assume that fish of a similar developmental stage are highly similar [30], so we will generate body models for different individuals than the tracked fish.

To calculate forces and torques, we assume that the fish has a constant density over its entire volume. In general, this is not the case—the presence of different types of tissue and a swim bladder cause an inhomogeneous mass distribution. The swim bladder causes the largest density difference within the body, since it is filled with gas. However, because it is located close to the fish’s centre of mass, it will have a small influence on the reconstructed torques. If necessary, density differences can be taken into account by creating a high-resolution tetrahedral mesh in the complete volume, on which a density distribution can be prescribed.

If needed, the current tracking method can be extended to track the complex deformation of the medial fin fold and the motion and deformation of the pectoral fins. This will require the addition of parameterised models of the medial and pectoral fins to the overall shape model. These parameters can be optimised simultaneously with the body deformation and motion. The image resolution of the data in the present work is insufficient to reliably perform this tracking, because we chose a large field of view to film extended swimming motions.

The method presented in this article is able to track a fish in three-dimensions by reconstructing its position, orientation (yaw, pitch and roll) and body curvature from high-speed video, with sufficient accuracy to compute resultant forces and torques. Body kinematics and dynamics at this high level of detail will help to pave the way for in-depth mechanistic analyses of the biomechanics of locomotion and manoeuvring in swimming fish.

## Supporting Information

S1 Appendix Full mathematical background of the tracking method. It describes the used equations in detail, and discusses how they were implemented.
(PDF)

S1 Video Example tracked movie. The example three-camera video sequence of a three days post fertilisation zebrafish larva shown in the article, overlayed with projections (in red) of the tracked fish model and its centreline (in green).
(MP4)

## References

[1] Hunter JR. Swimming and feeding behavior of larval anchovy *Engraulis mordax*. Fish Bull. 1972;70(82):1–834.

[2] Eloy C. Optimal Strouhal number for swimming animals. J Fluids Struct. 2012;30:205–218. 10.1016/j.jfluidstructs.2012.02.008

[3] Borazjani I, Sotiropoulos F. Numerical investigation of the hydrodynamics of carangiform swimming in the transitional and inertial flow regimes. J Exp Biol. 2008;211(10):1541–1558. 10.1242/jeb.015644 18456881

[4] Li G, Müller UK, Van Leeuwen JL, Liu H. Body dynamics and hydrodynamics of swimming fish larvae: a computational study. J Exp Biol. 2012;215(22):4015–4033. 10.1242/jeb.071837 23100489

[5] Liao JC, Beal DN, Lauder GV, Triantafyllou MS. The Kármán gait: novel body kinematics of rainbow trout swimming in a vortex street. J Exp Biol. 2003;206(6):1059–1073. 10.1242/jeb.00209 12582148

[6] Müller UK, Van Leeuwen JL. Swimming of larval zebrafish: ontogeny of body waves and implications for locomotory development. J Exp Biol. 2004;207(5):853–868. 10.1242/jeb.00821 14747416

[7] Green MH, Ho RK, Hale ME. Movement and function of the pectoral fins of the larval zebrafish (*Danio rerio*) during slow swimming. J Exp Biol. 2011;214(18):3111–3123. 10.1242/jeb.057497 21865524

[8] Tytell ED, Lauder GV. The C-start escape response of *Polypterus senegalus*: bilateral muscle activity and variation during stage 1 and 2. J Exp Biol. 2002;205(17):2591–2603. 12151365 10.1242/jeb.205.17.2591

[9] MacIver MA, Nelson ME. Body modeling and model-based tracking for neuroethology. J Neurosci Methods. 2000;95(2):133–143. 10.1016/S0165-0270(99)00161-2 10752484

[10] Kasapi MA, Domenici P, Blake RW, Harper D. The kinematics and performance of escape responses of the knifefish *Xenomystus nigri*. Can J Zool. 1993;71(1):189–195. 10.1139/z93-026

[11] Fontaine E, Lentink D, Kranenbarg S, Müller UK, Van Leeuwen JL, Barr AH, et al Automated visual tracking for studying the ontogeny of zebrafish swimming. J Exp Biol. 2008;211(8):1305–1316. 10.1242/jeb.010272 18375855

[12] Xiong G, Lauder GV. Center of mass motion in swimming fish: effects of speed and locomotor mode during undulatory propulsion. Zoology. 2014;117(4):269–281. 10.1016/j.zool.2014.03.002 24925455

[13] Butail S, Paley DA. Three-dimensional reconstruction of the fast-start swimming kinematics of densely schooling fish. J R Soc Interface. 2012;9(66):77–88. 10.1098/rsif.2011.0113 21642367 PMC3223621

[14] Koopman B, Grootenboer HJ, De Jongh HJ. An inverse dynamics model for the analysis, reconstruction and prediction of bipedal walking. J Biomech. 1995;28(11):1369–1376. 10.1016/0021-9290(94)00185-7 8522549

[15] Muijres FT, Elzinga MJ, Iwasaki NA, Dickinson MH. Body saccades of Drosophila consist of stereotyped banked turns. J Exp Biol. 2015;218(6):864–875. 10.1242/jeb.114280 25657212

[16] Van Leeuwen JL, Voesenek CJ, Müller UK. How body torque and Strouhal number change with swimming speed and developmental stage in larval zebrafish. J R Soc Interface. 2015;12(110):20150479 10.1098/rsif.2015.0479 26269230 PMC4614456

[17] Hess F, Videler JJ. Fast continuous swimming of saithe (*Pollachius virens*): a dynamic analysis of bending moments and muscle power. J Exp Biol. 1984;109(1):229–251.

[18] Cheng JY, Blickhan R. Bending moment distribution along swimming fish. Journal of Theoretical Biology. 1994;168(3):337–348. 10.1006/jtbi.1994.1114

[19] Li G, Müller UK, Van Leeuwen JL, Liu H. Escape trajectories are deflected when fish larvae intercept their own C-start wake. J R Soc Interface. 2014;11(101):20140848–20140848. 10.1098/rsif.2014.0848 25401174 PMC4223905

[20] Hughes NF, Kelly LH. New techniques for 3-D video tracking of fish swimming movements in still or flowing water. Can J Fish Aquat Sci. 1996;53(11):2473–2483. Available from: 10.1139/cjfas-53-11-2473. 10.1139/cjfas-53-11-2473

[21] Thorsen DH. Swimming of larval zebrafish: fin-axis coordination and implications for function and neural control. Journal of Experimental Biology. 2004;207(24):4175–4183. 10.1242/jeb.01285 15531638

[22] Nelder JA, Mead R. A simplex-method for function minimization. Comput J. 1965;7(4):308–313. 10.1093/comjnl/7.4.308

[23] Lagarias JC, Reeds JA, Wright MH, Wright PE. Convergence properties of the Nelder-Mead simplex method in low dimensions. SIAM J Optimiz. 1998;9(1):112–147. 10.1137/S1052623496303470

[24] Eilers PHC. A perfect smoother. Anal Chem. 2003;75(14):3631–3636. 10.1021/ac034173t 14570219

[25] Stickel JJ. Data smoothing and numerical differentiation by a regularization method. Comput Chem Eng. 2010;34(4):467–475. 10.1016/j.compchemeng.2009.10.007

[26] Dobrovolskis AR. Inertia of any polyhedron. Icarus. 1996;124(2):698–704. 10.1006/icar.1996.0243

[27] Simo J, Tarnow N, Doblare M. Non-linear dynamics of three-dimensional rods: exact energy and momentum conserving algorithms. Int J Numer Meth Eng. 1995;38(9):1431–1473. 10.1002/nme.1620380903

[28] Zupan E, Saje M. Integrating rotation from angular velocity. Adv Eng Softw. 2011;42(9):723–733. 10.1016/j.advengsoft.2011.05.010

[29] Van den Boogaart JGM, Muller M, Osse JWM. Structure and function of the median finfold in larval teleosts. J Exp Biol. 2012;215(14):2359–2368. 10.1242/jeb.065615 22723474

[30] Parichy DM, Elizondo MR, Mills MG, Gordon TN, Engeszer RE. Normal table of postembryonic zebrafish development: Staging by externally visible anatomy of the living fish. Dev Dynam. 2009;238(12):2975–3015. 10.1002/dvdy.22113 PMC3030279 19891001

