# Supplementary material for: Automated Reconstruction of Three-Dimensional Fish Motion, Forces, and Torques
Source: PLoS One. 2016 Jan 11;11(1):e0146682. doi: 10.1371/journal.pone.0146682 (PMC4713831; doi:10.1371/journal.pone.0146682)
Supplement: S1 Appendix — It describes the used equations in detail, and discusses how they were implemented. (PDF) [file pone.0146682.s001.pdf]

Automated Reconstruction of Three-dimensional Fish Motion, Forces, and Torques – Mathematical Background

Cees J. Voesenek[1]*, Remco P. M. Pieters[1], Johan L. van Leeuwen[1]

1 Experimental Zoology, Department of Animal Sciences, Wageningen University, Wageningen, Netherlands

*** cees.voesenek@wur.nl**

Creating the parameterised fish model

Shape

We describe the fish in its undeformed state in a coordinate system $(\hat{x}, \hat{y}, \hat{z})$ attached to the snout of the fish, with $\hat{x}$ in caudal-rostral direction, $\hat{y}$ in lateral direction and $\hat{z}$ in ventral-dorsal direction such that they form a right-handed coordinate system.

A (super-)elliptic cross-sectional shape is used for the eyes, the fin fold and the region of the body without yolk sack is given by:

$$\begin{cases} \hat{y}(\psi) = r_{\hat{y}} \cos(\psi); \\ \hat{z}(\psi) = \hat{z}_0 \pm r_{\hat{z}} \left[1 - \left(\frac{|\hat{y}(\psi)|}{r_{\hat{y}}} \right)^n \right]^{\frac{1}{n}}, \end{cases} \tag{1}$$

with $r_{\hat{y}}$ and $r_{\hat{z}}$ the radii in respectively $\hat{y}$- and $\hat{z}$-direction and $\psi = [0, 2\pi]$ where the sign in the equation for $\hat{z}$ depends on the considered quadrant: $+$ in the positive $\hat{z}$-quadrant, $-$ in the negative $\hat{z}$-quadrant. For the fin fold and body cross-sections in the tail, the exponent $n = 2$, making it a regular ellipse. The elliptic exponent for the eyes is 2.3.

The slices of the body with a bulging yolk sack have their egg-like cross-sectional shape defined by a cubic spline fit (using MATLAB's `spline`) through 4 points: $(\hat{x}_0, r_{\hat{y}}, \hat{z}_0 + h_{\text{shift}})$, $(\hat{x}_0, 0, \hat{z}_0 + r_{\hat{z}})$, $(\hat{x}_0, -r_{\hat{y}}, \hat{z}_0 + h_{\text{shift}})$, $(\hat{x}_0, 0, \hat{z}_0 - r_{\hat{z}})$. To prevent edge effects of the spline, we perform the fit on three repetitions of the points, and only use the middle section as a cross-section.

We build up the body with N_{lon} cross-sections and N_{circ} points along the circumference of each cross-section. We can connect each set of two cross-sections with $(N_{\text{lon}} - 1)N_{\text{circ}}$ quadrilateral faces, or $2(N_{\text{lon}} - 1)N_{\text{circ}}$ triangular faces. To close the surface, we cap the front- and aftmost cross-sections with N_{circ} triangular faces each.

Parameterisation

The body curvature κ is prescribed in a small number ($< N_{\text{lon}}$, 7 for the zebrafish larva) of control points along the length of the fish. We define a normalised parameter along the fish length $s = [0, 1]$ and use it to create a cubic spline (MATLAB's `spline`) that interpolates the curvature control points. We analytically integrate each piecewise polynomial of the curvature spline to obtain the local deformation angle:

$$\theta(s) = \ell \int_0^s \kappa(s)\, \mathrm{d}s, \tag{2}$$

with ℓ the body length. We model the fish's centreline as a sequence of straight segments i of length $\Delta\ell = \ell/(N_{\mathrm{lon}} - 1)$, and apply the local deformation angle to each:

$$\hat{x}_{\mathrm{centre},i} = \begin{cases} 0 & i = 1 \\ \hat{x}_{i-1} - \Delta\ell \cos\left(\frac{\theta_{i-1}+\theta_i}{2}\right) & \text{otherwise} \end{cases}$$
$$\hat{y}_{\mathrm{centre},i} = \begin{cases} 0 & i = 1 \\ \hat{y}_{i-1} + \Delta\ell \sin\left(\frac{\theta_{i-1}+\theta_i}{2}\right) & \text{otherwise} \end{cases} \tag{3}$$

in coordinates relative to the fish's head. Every cross-section i consists of N_{circ} circumferential points to which we assign the index j. We rotate every point i, j on the cross-section with the rotation of the centreline, such that it remains perpendicular:

$$\hat{\mathbf{x}}_{\mathrm{def},i,j} = \begin{pmatrix} \hat{x}_{\mathrm{def},i,j} \\ \hat{y}_{\mathrm{def},i,j} \\ \hat{z}_{\mathrm{def},i,j} \end{pmatrix} = \begin{pmatrix} \hat{x}_{\mathrm{centre},i} + \hat{y}_{i,j} \sin\theta_i \\ \hat{y}_{\mathrm{centre},i} + \hat{y}_{i,j} \cos\theta_i \\ \hat{z}_{i,j} \end{pmatrix} \tag{4}$$

We now have a deformed fish in the $(\hat{x}, \hat{y}, \hat{z})$ coordinate system, which we place in world coordinates with the position $\mathbf{x}_{\mathrm{snout}}$ and rotation $\boldsymbol{\varphi}$ at the snout. The angles φ_{roll}, φ_{pitch}, φ_{yaw} (abbreviated as φ_{r}, φ_{p}, φ_{y} for reasons of clarity) specifying the rotation are converted to a rotation matrix:

$$\mathrm{R} = \begin{bmatrix} \cos\varphi_{\mathrm{p}}\cos\varphi_{\mathrm{y}} & -\cos\varphi_{\mathrm{p}}\sin\varphi_{\mathrm{y}} & \sin\varphi_{\mathrm{p}} \\ \begin{pmatrix} \sin\varphi_{\mathrm{r}}\sin\varphi_{\mathrm{p}}\cos\varphi_{\mathrm{y}}+ \\ \cos\varphi_{\mathrm{r}}\sin\varphi_{\mathrm{y}} \end{pmatrix} & \begin{pmatrix} -\sin\varphi_{\mathrm{r}}\sin\varphi_{\mathrm{p}}\sin\varphi_{\mathrm{y}}+ \\ \cos\varphi_{\mathrm{r}}\cos\varphi_{\mathrm{y}} \end{pmatrix} & -\sin\varphi_{\mathrm{r}}\cos\varphi_{\mathrm{p}} \\ \begin{pmatrix} -\cos\varphi_{\mathrm{r}}\sin\varphi_{\mathrm{p}}\cos\varphi_{\mathrm{y}}+ \\ \sin\varphi_{\mathrm{r}}\sin\varphi_{\mathrm{y}} \end{pmatrix} & \begin{pmatrix} \cos\varphi_{\mathrm{r}}\sin\varphi_{\mathrm{p}}\sin\varphi_{\mathrm{y}}+ \\ \sin\varphi_{\mathrm{r}}\cos\varphi_{\mathrm{y}} \end{pmatrix} & \cos\varphi_{\mathrm{r}}\cos\varphi_{\mathrm{p}} \end{bmatrix} \tag{5}$$

We compute the final position of each point on the fish with:

$$\mathbf{x}_{\mathrm{def},i,j} = \mathrm{R}\hat{\mathbf{x}}_{\mathrm{def},i,j} + \mathbf{x}_{\mathrm{snout}}. \tag{6}$$

Finally, all points are reassembled into a three-dimensional surface of quadrilaterals during tracking and of triangles during post-processing.

Fitting the body model

Objective function

The objective function that we aim to minimise in order to achieve an optimal fit is defined as follows:

$$f_{\mathrm{tot}}(\boldsymbol{\Omega}) = f_{\mathrm{GoF}}(\boldsymbol{\Omega}) + f_{\mathrm{reg}}(\boldsymbol{\Omega}). \tag{7}$$

Here, $\boldsymbol{\Omega}$ is the list of optimised parameters, $f_{\mathrm{GoF}}(\boldsymbol{\Omega})$ is a term indicating how accurately the fish model approaches the high-speed video images, and $f_{\mathrm{reg}}(\boldsymbol{\Omega})$ is a term that penalises "unsmoothness". The two terms are discussed in detail below.

Goodness of fit

To compare the virtual images to the segmented high-speed video images, we project the three-dimensional shape of the fish onto virtual cameras. We define a left-handed camera coordinate system (ξ, η, ϑ), respectively rows, columns, and a coordinate perpendicular to the image plane in the viewing direction of the camera. A transformation matrix from camera to world coordinate system is given by:

$$\mathrm{T}_{\mathrm{cam}} = \begin{bmatrix} \omega_{x,\xi} & \omega_{x,\eta} & \omega_{x,\vartheta} \\ \omega_{y,\xi} & \omega_{y,\eta} & \omega_{y,\vartheta} \\ \omega_{z,\xi} & \omega_{z,\eta} & \omega_{z,\vartheta} \end{bmatrix} \tag{8}$$

Since we are considering a camera system with parallel light, the distance of an object from the camera has no influence on its projected size. The light is collimated to within 10° divergence, so perspective effects are negligible at the millimetre scales of zebrafish motion. Projecting objects onto the camera is therefore only a coordinate transformation to camera coordinates (i.e., a rotation, translation and rescaling):

$$\boldsymbol{\xi} = \frac{1}{d_{\text{scale}}} \mathrm{T}_{\text{cam}}^{\mathrm{T}} (\mathbf{x}_{\text{obj}} - \mathbf{x}_{\text{cam}}) + 1, \tag{9}$$

where $\mathbf{x}_{\text{object}}$ are the object coordinates, $\mathbf{x}_{\text{cam}}$ the camera coordinate system origin (i.e., the top left corner of the image), d_{scale} a scaling factor in unit distance per pixel. The 1 is added because by convention, the top left corner is denoted as having row-column (i.e., ξ, η) coordinates $(1, 1)$. Because the transformation matrix T_{cam} is orthogonal, we can invert the matrix by taking its transpose. The image plane coordinates of the projected point are given by (ξ, η); the third coordinate ϑ can be ignored.

When considering cases with significant magnification effects (e.g., when no collimated light setup is used), we use a simple linear pinhole model, where image magnification is linear with distance from the camera. In that case, we use the ϑ coordinate to determine the scaling factor for the current projected point and thus compute its projection on the image plane.

To compute the goodness of fit, we project the three-dimensional surface of the fish onto the camera plane using the procedure outlined above. We then overlay the fish silhouette in image coordinates onto the segmented high-speed video frames. All pixels in the combined image are counted, and the pixels that overlap between the model fish and the actual fish are subtracted from this number. The final result is the number of mismatching pixels – an expression for the goodness of fit.

Penalty function

The regularising term in the objective function is of the form

$$f_{\text{reg}} = \int_0^1 w(s) \left(\frac{\mathrm{d}\kappa(s)}{\mathrm{d}s} \right)^2 \, \mathrm{d}s, \tag{10}$$

where $w(s)$ is a weighting function that allows stronger suppression of curvature near the head than near the tail. For the zebrafish, we prescribe $w(s)$ as a cubic spline fit (MATLAB's `spline`) in logarithmic space of control points along the body. In our case of the larval zebrafish, we prescribe two points at $s = \{0, 1\}$. Since a cubic spline fit of two points is linear, the result is a function of the form $w(s) = c_0 e^{-c_1 s}$.

Initialisation

The initial set of model parameters for the first frame $\boldsymbol{\omega_0}$ is computed from two user-clicked points per camera: the snout and the tail. Rewriting Eqn. 9 gives, for every camera,

$$\frac{1}{d_{\text{scale}}} \mathrm{T}_{\text{cam}}^{\mathrm{T}} \mathbf{x}_{\text{obj}} = \boldsymbol{\xi} + \frac{1}{d_{\text{scale}}} \mathrm{T}_{\text{cam}}^{\mathrm{T}} \mathbf{x}_{\text{cam}} - 1. \tag{11}$$

Manually indicating a point in an image will give us the first two components of $\boldsymbol{\xi}$, so we can use the first two equations of this system for each camera. This results in an overdetermined linear system (for $N_{\text{cam}} > 2$): we have 3 unknowns (x, y, z) and $2N_{\text{cam}}$ equations. We solve this system with least-squares (MATLAB's \ operator) to obtain the snout and tail positions in 3D.

The snout position can be directly used to initialise the position parameter for the optimisation. We set the initial roll angle to 0, the pitch angle to

$$\varphi_{\text{pitch}} = \arctan \left(\frac{z_{\text{snout}} - z_{\text{tail}}}{\left[(x_{\text{snout}} - x_{\text{tail}})^2 + (y_{\text{snout}} - y_{\text{tail}})^2 \right]^{\frac{1}{2}}} \right) \tag{12}$$

and the yaw angle to

$$\varphi_{\mathrm{yaw}} = \arctan\left(\frac{y_{\mathrm{snout}} - y_{\mathrm{tail}}}{x_{\mathrm{snout}} - x_{\mathrm{tail}}}\right). \tag{13}$$

The arctangents are computed using MATLAB's `atan2` function, that takes into account the sign of the nominator and denominator to compute the angle in the correct quadrant.

We approximate the fish length ℓ as follows:

$$\ell_{\mathrm{init}} = \sqrt{(x_{\mathrm{snout}} - x_{\mathrm{tail}})^2 + (y_{\mathrm{snout}} - y_{\mathrm{tail}})^2 + (z_{\mathrm{snout}} - z_{\mathrm{tail}})^2} \tag{14}$$

This approximation is used as an initial condition for an optimisation in the first frame of the sequence, where we allow the fish length to vary. The final, optimised fish length ℓ is then kept fixed in the rest of the video sequence.

Solution extrapolation

We extrapolate the solution from previous time steps to initialise the next time step. We use the following:

$$\mathbf{\Omega}_{\mathrm{init},i_t} = \begin{cases} \mathbf{\Omega}_0 & i_t = 1 \\ \mathbf{\Omega}_{\mathrm{opt},i_t-1} & i_t = 2 \\ -\mathbf{\Omega}_{\mathrm{opt},i_t-2} + 2\mathbf{\Omega}_{\mathrm{opt},i_t-1} & i_t = 3 \\ \mathbf{\Omega}_{\mathrm{opt},i_t-3} - 3\mathbf{\Omega}_{\mathrm{opt},i_t-2} + 3\mathbf{\Omega}_{\mathrm{opt},i_t-1} & i_t > 3 \end{cases} \tag{15}$$

where $i_t \in \mathbb{N}$ denotes the time step, $\mathbf{\Omega}_{\mathrm{init},i_t}$ is the set of parameters used to initialise the optimisation at time step i_t and $\mathbf{\Omega}_{\mathrm{init},i_t}$ is the final, optimised set of parameters at time step i_t.

Post-processing and inverse dynamics

Smoothing

In general, the solution from the optimisation procedure is insufficiently smooth in time to accurately compute (second) derivatives. We perform *post hoc* smoothing to remove spurious high-frequency components, but retain (most of) the physical signal. We use a regularised data fit similar to [1], where smoothing is presented as a minimisation problem:

$$\underset{\phi_{\mathrm{sm}}}{\arg\min} \left\{ \int_0^{t_{\max}} \left|\phi_{\mathrm{data}}(t) - \phi_{\mathrm{sm}}(t)\right|^2 \,\mathrm{d}t \; + \lambda_{\mathrm{sm}} \int_0^{t_{\max}} \left|\frac{\mathrm{d}^n \phi_{\mathrm{sm}}(t)}{\mathrm{d}t^n}\right|^2 \,\mathrm{d}t \right\}, \tag{16}$$

where ϕ_{sm} are the smoothed data points, ϕ_{data} are the measured data points, λ_{sm} is a parameter controlling the amount of regularisation and n is the order of the derivative used for the penalty function. It has been shown [1] that the solution for a discrete number of points is given by

$$\phi_{\mathrm{sm}} = (\mathrm{I} + \lambda_{\mathrm{sm}} \mathrm{D}^{\mathrm{T}} \mathrm{D})^{-1} \phi_{\mathrm{data}}, \tag{17}$$

where D is a matrix that approximates the required n^{th}-order derivative. We assume here that our fitted points are located at the same time instants as the data points. For the D matrix, we use 4 subsequent one-sided differences (using MATLAB's `diff`) to approximate the fourth order derivative, ensuring that our second derivatives are smooth.

To combat edge effects of the smoothing, we separate two cases: starts, where the animal accelerates from a stationary position, and "continuous" swimming, where it enters and exits the field of view while swimming. In the case of starts, we know that the animal is stationary before the time series starts. We therefore add 25 time points before $t = 0$ which all have the value at $t = 0$, thus forcing the solution to have a realistic zero gradient at the start. In the case of "continuous" swimming, we can simply cut off the first few (in our case 5) points, where the edge effects are strongest. For both cases, we cut off the same number of points off the end of the time series.

Derivatives

Differentiation is required to compute velocity and acceleration from the centre of mass position, and torque from the angular momentum vector. We use the same procedure for any time series, for first derivatives:

$$\frac{\partial \phi}{\partial t} \approx \begin{cases} \frac{-3\phi_1+4\phi_2-\phi_3}{2\Delta t} & i_t = 1 \\ \frac{-\phi_{i_t-1}+\phi_{i_t+1}}{2\Delta t} & 1 < i_t < N_t \\ \frac{\phi_{N_t-2}-4\phi_{N_t-1}+3\phi_{N_t}}{2\Delta t} & i_t = N_t \end{cases} \tag{18}$$

for second derivatives:

$$\frac{\partial^2 \phi}{\partial t^2} \approx \begin{cases} \frac{\phi_1-2\phi_2+\phi_3}{\Delta t^2} & i_t = 1 \\ \frac{\phi_{i_t-1}-2\phi_{i_t}+\phi_{i_t+1}}{\Delta t^2} & 1 < i_t < N_t \\ \frac{\phi_{N_t-2}-2\phi_{N_t-1}+\phi_{N_t}}{\Delta t^2} & i_t = N_t \end{cases} \tag{19}$$

Resultant forces and torques

The resultant force $\mathbf{F}_{\text{net}}$ on the fish can be computed from Newton's second law:

$$\mathbf{F}_{\text{net}} = m\mathbf{a}_{\text{CoM}} = \rho V \frac{\mathrm{d}^2\mathbf{r}_{\text{CoM}}}{\mathrm{d}t^2}, \tag{20}$$

where m is fish mass, ρ is fish density, and $\mathbf{a}_{\text{CoM}}$ denotes the acceleration of the centre of mass; the volume V and position of the centre of mass $\mathbf{r}_{\text{CoM}}$ can be computed using the method by [2] from the triangulated surface of the body model. Every triangular face is made into a tetrahedron by taking the coordinate system origin as a fourth vertex, see Fig 1A. Calculations can be performed separately on each of these tetrahedra, and subsequently summed to yield the result for the complete fish.

The method by [2] only computes the centre of mass and the moment of inertia matrix; we extend their method to compute angular momentum for an arbitrary triangulated polyhedron of constant density. Angular momentum is defined as:

$$\mathbf{L} = \rho \iiint\limits_V \mathbf{r}^*(\mathbf{x}) \times \mathbf{v}^*(\mathbf{x})\, \mathrm{d}V, \tag{21}$$

where the asterisks denotes quantities measured with respect to the centre of mass. The integrand is linear, so we can evaluate Eqn. 21 for each tetrahedron separately and then sum the results. To simplify this computation, we transform every tetrahedron into a unit tetrahedron in the origin of a new coordinate system (x', y', z'), see Fig 1B. This is a linear transformation, with the following transformation matrix from unit to world tetrahedron:

$$\mathrm{T}_{\text{tet}} = \begin{bmatrix} x^*_{d,k} & x^*_{e,k} & x^*_{f,k} \\ y^*_{d,k} & y^*_{e,k} & y^*_{f,k} \\ z^*_{d,k} & z^*_{e,k} & z^*_{f,k} \end{bmatrix} \tag{22}$$

The transformed integral for every tetrahedron becomes

$$\Delta\mathbf{L} = \rho \det \mathrm{T}_{\text{tet}} \int_0^1 \int_0^{1-x'} \int_0^{1-x'-y'} \mathbf{r}^*(\mathbf{x}') \times \mathbf{v}^*(\mathbf{x}')\; \mathrm{d}z'\mathrm{d}y'\mathrm{d}x', \tag{23}$$

Transformed to a unit tetrahedron, linear interpolation of quantities known at its vertices reduces to a single matrix multiplication. The interpolating matrices for the position and velocity are given by:

$$\Gamma_r = \begin{bmatrix} x^*_{d,k} & x^*_{e,k} & x^*_{f,k} \\ y^*_{d,k} & y^*_{e,k} & y^*_{f,k} \\ z^*_{d,k} & z^*_{e,k} & z^*_{f,k} \end{bmatrix} \tag{24}$$

$$\Gamma_v = \begin{bmatrix} v^*_{x,d,k} & v^*_{x,e,k} & v^*_{x,f,k} \\ v^*_{y,d,k} & v^*_{y,e,k} & v^*_{y,f,k} \\ v^*_{z,d,k} & v^*_{z,e,k} & v^*_{z,f,k} \end{bmatrix} \tag{25}$$

With interpolation, Eqn. 23 can be written as:

$$\Delta\mathbf{L}_k = \rho \det \mathrm{T}_{\mathrm{tet}} \int_0^1 \int_0^{1-x'} \int_0^{1-x'-y'} \Gamma_r \mathbf{x}' \times \Gamma_v \mathbf{x}' \, \mathrm{d}z' \mathrm{d}y' \mathrm{d}x'. \tag{26}$$

Evaluating this integral (using Maple 18, Maplesoft, Waterloo, Ontario, Canada) gives the following expressions for the x-, y- and z-components of the angular momentum vector:

$$\begin{aligned} \Delta L_{x,k} = \tfrac{\rho \Delta V_k}{20} \big[& + y^*_{d,k} \, (2v^*_{z,d,k} + v^*_{z,e,k} + v^*_{z,f,k}) \\ & + y^*_{e,k} \, (v^*_{z,d,k} + 2v^*_{z,e,k} + v^*_{z,f,k}) \\ & + y^*_{f,k} \, (v^*_{z,d,k} + v^*_{z,e,k} + 2v^*_{z,f,k}) \\ & - z^*_{d,k} \, (2v^*_{y,d,k} + v^*_{y,e,k} + v^*_{y,f,k}) \\ & - z^*_{e,k} \, (v^*_{y,d,k} + 2v^*_{y,e,k} + v^*_{y,f,k}) \\ & - z^*_{f,k} \, (v^*_{y,d,k} + v^*_{y,e,k} + 2v^*_{y,f,k}) \big]; \end{aligned} \tag{27}$$

$$\begin{aligned} \Delta L_{y,k} = \tfrac{\rho \Delta V_k}{20} \big[& - x^*_{d,k} \, (2v^*_{z,d,k} + v^*_{z,e,k} + v^*_{z,f,k}) \\ & - x^*_{e,k} \, (v^*_{z,d,k} + 2v^*_{z,e,k} + v^*_{z,f,k}) \\ & - x^*_{f,k} \, (v^*_{z,d,k} + v^*_{z,e,k} + 2v^*_{z,f,k}) \\ & + z^*_{d,k} \, (2v^*_{x,d,k} + v^*_{x,e,k} + v^*_{x,f,k}) \\ & + z^*_{e,k} \, (v^*_{x,d,k} + 2v^*_{x,e,k} + v^*_{x,f,k}) \\ & + z^*_{f,k} \, (v^*_{x,d,k} + v^*_{x,e,k} + 2v^*_{x,f,k}) \big]; \end{aligned} \tag{28}$$

$$\begin{aligned} \Delta L_{z,k} = \tfrac{\rho \Delta V_k}{20} \big[& + x^*_{d,k} \, (2v^*_{y,d,k} + v^*_{y,e,k} + v^*_{y,f,k}) \\ & + x^*_{e,k} \, (v^*_{y,d,k} + 2v^*_{y,e,k} + v^*_{y,f,k}) \\ & + x^*_{f,k} \, (v^*_{y,d,k} + v^*_{y,e,k} + 2v^*_{y,f,k}) \\ & - y^*_{d,k} \, (2v^*_{x,d,k} + v^*_{x,e,k} + v^*_{x,f,k}) \\ & - y^*_{e,k} \, (v^*_{x,d,k} + 2v^*_{x,e,k} + v^*_{x,f,k}) \\ & - y^*_{f,k} \, (v^*_{x,d,k} + v^*_{x,e,k} + 2v^*_{x,f,k}) \big]. \end{aligned} \tag{29}$$

The total angular momentum is then given by the sum over all tetrahedra k:

$$\mathbf{L} = \sum_k \Delta\mathbf{L}_k. \tag{30}$$

With the angular momentum known in each time step, we can compute the net torque by taking its time derivative:

$$\boldsymbol{\tau}_{\mathrm{net}} = \frac{\mathrm{d}\mathbf{L}}{\mathrm{d}t}. \tag{31}$$

Fish reference system

To make interpretation of the forces and torques more intuitive, we transform them to a local fish coordinate system $(x_{\mathrm{fish}}, y_{\mathrm{fish}}, z_{\mathrm{fish}})$ that rotates with the changing orientation of the fish. To create this fish axis system, we first rotate the world coordinate system so it is aligned with the fish's head, using the rotation matrix in Eqn. 5. This transforms the fish to the $(\hat{x}, \hat{y}, \hat{z})$ coordinate system defined in section Creating the parameterised fish model, where the $\hat{x}, \hat{y}$-plane is aligned with the deformation plane of the fish, and $\hat{z}$ is perpendicular to it. The $\hat{x}$-coordinate is aligned with the head, which rotates with a large amplitude and makes interpretation of the time-variation of the forces and torques difficult.

In order to define an angle that is related to the entire body, we use a "body angle" [3], defined as:

$$\varphi_{\mathrm{body}} = \frac{\int_0^1 I_{\mathrm{CoM},i}(s)\alpha(s) \, \mathrm{d}s}{\int_0^1 I_{\mathrm{CoM},i}(s) \, \mathrm{d}s}, \tag{32}$$

where $I_{\mathrm{CoM},i}(s)$ is the moment of inertia about the centre of mass per unit length of the cross-section at s and $\alpha(s)$ is the angle between a line projected from the centre of mass to the centre of segment and horizontal. The body angle is effectively a moment of inertia-weighted average of the local angles of each segment.

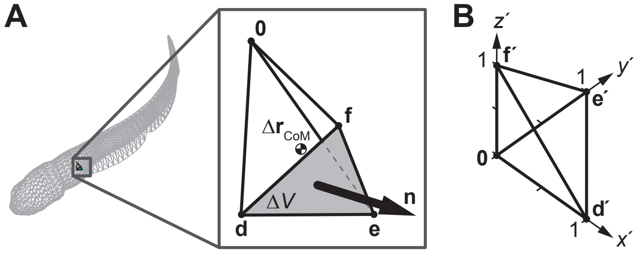

Figure 1. Tetrahedra used for computation of derived quantities: centre of mass position and instantaneous forces and torques. (A) To compute volume properties from a surface description, a tetrahedron is created for each surface triangle **d e f**, with a volume ΔV and a centre of mass position $\Delta\mathbf{r}_{\text{CoM}}$. The sign of its contribution is determined by the direction of the normal **n**. (B) The same tetrahedron is transformed into a unit tetrahedron for easier interpolation and integration, allowing us to compute the contribution of each tetrahedron to the angular momentum separately with a single analytical expression as a function of the values at the vertices.

We can express the local moment of inertia per unit length as the contribution of the cross-section about its own vertical centre axis $I_{\text{cross},i}$ and its contribution with respect to the centre of mass

$$I_{\text{CoM},i} = I_{\text{cross},i} + \mu r^2_{\text{CoM},i}, \tag{33}$$

where μ is a mass per unit length and $r^2_{\text{CoM},i}$ the distance between the centre of mass in the $x_{\text{fish}}, y_{\text{fish}}$-plane and the cross-section centre. We compute the moment of inertia per unit length and area, and thus mass, assuming constant density, of each cross-section with the expressions for arbitrary polygons [4]. The local segment angle α is computed by projecting a line from the centre of mass to the segment centre in the deformation plane (i.e., in $\hat{x}, \hat{y}, \hat{z}$-coordinates), and computing the angle it makes with respect to the head:

$$\alpha_i = \arctan\left(\frac{\hat{y}_{\text{centre},i} - \hat{y}_{\text{CoM}}}{\hat{x}_{\text{centre},i} - \hat{x}_{\text{CoM}}}\right), \tag{34}$$

making sure we get the correct sign depending on the quadrant we are in by using MATLAB's `atan2` function.

We compute the body angle by evaluating the integral from Eqn. 32 using MATLAB's `trapz` function. This results in a series of body angles over time that may still have discontinuities where the centre of mass crosses the centreline; in these points, the angle "reflects" around an unknown line since all the projected lines flip direction. To remove these discontinuities, we average a one-sided derivative left and right of the discontinuity. We then extrapolate the solution from the point left of the discontinuity:

$$\alpha_i = \alpha_{i-1} + \frac{1}{2}\left(\frac{\alpha_{i-3} - 4\alpha_{i-2} + 3\alpha_{i-1}}{2} + \frac{-3\alpha_i + 4\alpha_{i+1} - \alpha_{i+2}}{2}\right), \tag{35}$$

where i is the index of the point just right of the discontinuity. The entire sequence to the right of the discontinuity is shifted, retaining gradient information. We iterate over all discontinuities in the sequence, yielding a body angle that is continuous from the first angle onwards. Since we start with a straight fish in the $\hat{x}, \hat{y}, \hat{z}$-coordinates, we define this first angle as 0.

We rotate the head-aligned coordinate system along its vertical axis by the computed body angle. We represent the rotation as an axis and angle: respectively $\boldsymbol{\omega}_{z_{\text{head}}}$ (abbreviated in Eqn. 36 as $\boldsymbol{\omega}$), the unit orientation vector of the z_{head}-axis and φ_{body} (abbreviated in Eqn. 36 as φ).This results in a rotation matrix of the following form:

$$\text{R}_{\text{body}} = \begin{bmatrix} \begin{pmatrix} \cos\varphi \\ +\omega_x^2(1-\cos\varphi) \end{pmatrix} & \begin{pmatrix} \omega_x\omega_y(1-\cos\varphi) \\ -\omega_z\sin\varphi \end{pmatrix} & \begin{pmatrix} \omega_x\omega_z(1-\cos\varphi) \\ +\omega_y\sin\varphi \end{pmatrix} \\ \begin{pmatrix} \omega_x\omega_y(1-\cos\varphi) \\ +\omega_z\sin\varphi \end{pmatrix} & \begin{pmatrix} \cos\varphi \\ +\omega_y^2(1-\cos\varphi) \end{pmatrix} & \begin{pmatrix} \omega_y\omega_z(1-\cos\varphi) \\ -\omega_x\sin\varphi \end{pmatrix} \\ \begin{pmatrix} \omega_x\omega_z(1-\cos\varphi) \\ -\omega_y\sin\varphi \end{pmatrix} & \begin{pmatrix} \omega_y\omega_z(1-\cos\varphi) \\ +\omega_x\sin\varphi \end{pmatrix} & \begin{pmatrix} \cos\varphi \\ +\omega_z^2(1-\cos\varphi) \end{pmatrix} \end{bmatrix} \tag{36}$$

Applying this rotation matrix to the axes of the head coordinate system yields our final fish coordinate system $(x_{\mathrm{fish}}, y_{\mathrm{fish}}, z_{\mathrm{fish}})$, in which we express our forces and torques. We transform the force vector by projecting it onto each unit orientation vector of the axes, e.g.,

$$F_{x,\mathrm{fish}} = F_x \cdot \boldsymbol{\omega}_{x_{\mathrm{fish}}}. \tag{37}$$

We define an additional axis in the direction of the velocity vector:

$$\boldsymbol{\omega}_{\mathrm{fwd}} = \mathbf{v}_{\mathrm{CoM}}/|\mathbf{v}_{\mathrm{CoM}}|, \tag{38}$$

where $\mathbf{v}_{\mathrm{CoM}}$ is the instantaneous velocity vector of the centre of mass. We project the force to this axis to have an indication of the effective force propelling the fish.

References

1. Stickel JJ. Data smoothing and numerical differentiation by a regularization method. Comput Chem Eng. 2010;34(4):467–475. Available from: http://dx.doi.org/10.1016/j.compchemeng.2009.10.007.

2. Dobrovolskis AR. Inertia of any polyhedron. Icarus. 1996;124(2):698–704. Available from: http://dx.doi.org/10.1006/icar.1996.0243.

3. Van Leeuwen JL, Voesenek CJ, Müller UK. How body torque and Strouhal number change with swimming speed and developmental stage in larval zebrafish. J R Soc Interface. 2015;12(110):20150479. Available from: http://dx.doi.org/10.1098/rsif.2015.0479.

4. Liggett JA. Exact formulae for areas, volumes and moments of polygons and polyhedra. Commun Appl Numer M. 1988;4(6):815–820. Available from: http://dx.doi.org/10.1002/cnm.1630040616.
